# A Comprehensive Review of Low-Dose Interleukin-2 (IL-2) Therapy for Systemic Lupus Erythematosus: Mechanisms, Efficacy, and Clinical Applications

**DOI:** 10.7759/cureus.68748

**Published:** 2024-09-05

**Authors:** Amna Farooq, Shubam Trehan, Gurjot Singh, Nirav Arora, Tejal Mehta, Prateek Jain, Gaurav Bector, Aayush Jain, Rajpreet S Arora, Piyush Puri

**Affiliations:** 1 Internal Medicine, Yale School of Medicine, Waterbury, USA; 2 Internal Medicine, Dayanand Medical College and Hospital, Ludhiana, IND; 3 Internal Medicine, Maharaj Sawan Singh (MSS) Charitable Hospital, Beas, IND; 4 Computer Science, Lamar University, Beaumont, USA; 5 Internal Medicine, Dayanand Medical College and Hospital, Ludhiana , IND

**Keywords:** cytokine, il-2, low dose il-2, sle, sle treatment

## Abstract

Systemic lupus erythematosus (SLE) is a chronic autoimmune disease that causes extensive inflammation and tissue destruction across several organs. Conventional therapies, such as nonsteroidal anti-inflammatory drugs (NSAIDs), corticosteroids, and immunosuppressive drugs, can have serious adverse effects and are not always successful. This study looks at the possibility of low-dose interleukin-2 (IL-2) therapy as a new treatment for SLE, focusing on its mechanics, effectiveness, and clinical applicability.

Low-dose IL-2 treatment selectively increases and activates regulatory T cells (Tregs), which are essential for immunological tolerance but are often lacking in SLE patients. Unlike standard medicines, which widely inhibit the immune system, low-dose IL-2 provides a more tailored approach with fewer side effects. We examined preclinical and clinical research and discovered that low-dose IL-2 dramatically enhances Treg numbers and function, lowers disease activity, and improves clinical outcomes. The primary molecular processes include the stimulation of the Janus kinase - signal transducer of activators of transcription (JAK-STAT), phosphatidylinositol 3-kinase - protein kinase B (PI3K-Akt), and mitogen‑activated protein kinase (MAPK) pathways, which enhance Treg proliferation, survival, and activity. A thorough review of clinical studies finds that low-dose IL-2 treatment is well-tolerated and effective, with fewer side effects than biologics like belimumab and rituximab.

Furthermore, IL-2 therapy provides prospects for combination therapies, which may improve therapeutic success by addressing numerous components of the immune response. Despite these encouraging findings, problems such as patient response variability and the need for long-term safety data persist. Future research should prioritize refining dose regimes, discovering biomarkers for patient selection, and investigating combination medicines. Addressing these issues might solidify low-dose IL-2 treatment as a cornerstone in SLE care, providing a more accurate and individualized approach to immune regulation while considerably improving patient outcomes.

## Introduction and background

Systemic lupus erythematosus (SLE) is an intricate and persistent autoimmune illness marked by extensive inflammation and tissue harm that impacts many organs, such as the skin, joints, kidneys, brain, and other organs. The development of SLE is caused by a combination of genetic predisposition, environmental stimuli, and hormonal factors. This leads to the activation of T and B cells that respond against the body's own cells and the generation of autoantibodies [1]. Although there have been improvements in comprehending the cause of the disease, the management of SLE continues to be difficult because of its diverse character and wide range of clinical symptoms.

SLE mostly impacts women, especially those in their reproductive years, with a female-to-male ratio of around 9:1 [2]. The occurrence and frequency of SLE differ worldwide, with elevated rates observed in specific ethnic populations such as African Americans, Hispanics, and Asians [3]. The estimated frequency of Systemic Lupus Erythematosus (SLE) in the United States ranges from 20 to 150 cases per 100,000 persons, with notable variations depending on racial and ethnic origins [4]. On a global scale, the prevalence varies between 30 and 50 per 100,000 persons [5]. The burden of SLE is significant, not only because it is a long-lasting condition that requires lifetime therapy but also because it has a negative effect on quality of life, illness rates, and death rates. Individuals diagnosed with systemic lupus erythematosus (SLE) are at an elevated risk of developing cardiovascular disease, infections, and renal problems. These factors lead to higher healthcare consumption and expenses [6].

The primary objective of managing SLE is to effectively regulate disease activity, mitigate the occurrence of flares, and reduce any potential harm to organs. Common treatment protocols consist of nonsteroidal anti-inflammatory medications (NSAIDs), antimalarials (e.g., hydroxychloroquine), corticosteroids, and immunosuppressive medicines (e.g., azathioprine, mycophenolate mofetil, and cyclophosphamide) [7]. Although these medications can effectively manage symptoms and avoid flares, they are frequently accompanied by substantial side effects and long-term consequences. Corticosteroids are associated with a higher likelihood of developing osteoporosis, diabetes, and cardiovascular disease [8]. Immunosuppressive medications can heighten the susceptibility to infections and cancers [9].

Moreover, the diverse nature of systemic lupus erythematosus (SLE) presents a difficulty in customizing therapy for each patient. The condition exhibits significant variability in its manifestation, intensity, and reaction to treatment, thereby requiring an individualized approach to its care. Nevertheless, the absence of dependable biomarkers to forecast disease activity and treatment response adds complexity to this endeavor [10]. In addition, even with intensive immunosuppressive treatment, a considerable number of patients still suffer from ongoing disease activity and gradual deterioration of organ function [11]. This highlights the necessity for innovative treatment approaches that can better regulate the immune system and offer sustained management of disease activity with fewer adverse effects.

Recently, there has been substantial advancement in the development of novel therapeutic alternatives for SLE. Biologic treatments, which selectively target distinct elements of the immune system, have demonstrated potential in clinical studies. Belimumab, a monoclonal antibody that blocks B-cell activating factor (BAFF), was the initial biologic to receive approval for the treatment of SLE. Clinical trials have demonstrated its efficacy in reducing disease activity and flares [12]. Rituximab, a monoclonal antibody that specifically targets CD20 on B cells, has been utilized off-label in the treatment of SLE with varying degrees of effectiveness [13]. Recently, anifrolumab, an antibody that targets the interferon-alpha receptor, has been approved as a treatment option for individuals with SLE, especially those with moderate to severe disease activity [14]. However, even with these developments, the effectiveness of biologic therapy might vary, and not all patients are able to obtain sufficient control of their condition. In addition, these treatments can be costly and are linked to their own array of possible adverse effects, such as an elevated susceptibility to infections [15]. Hence, there is still a want for more therapy alternatives that can offer efficient and long-lasting management of SLE while maintaining a good safety record.

Interleukin-2 (IL-2) is a vital cytokine involved in immune system control. It enhances the development, viability, and activity of T cells, particularly regulatory T cells (Tregs), which play a crucial role in preserving immunological tolerance and avoiding autoimmune reactions [16]. In SLE, there is frequently an inequity between effector T cells and regulatory T cells (Tregs), with a relative insufficiency of Tregs playing a role in the breakdown of immunological tolerance and the emergence of autoimmunity [17]. Low-dose IL-2 treatment is a new method for regulating the immune system in SLE. The goal of low-dose IL-2 treatment is to restore immunological balance and induce tolerance by selectively increasing and activating Tregs. This strategy relies on the recognition that Tregs have elevated levels of the IL-2 receptor (IL-2R) and may be specifically amplified by administering small amounts of IL-2 without substantially activating effector T cells. In contrast, greater doses of IL-2 are necessary to activate effector T cells [18]. Preclinical investigations and initial clinical trials have shown evidence that supports the effectiveness and safety of low-dose IL-2 treatment in systemic lupus erythematosus (SLE). Studies conducted on mice with lupus have demonstrated that administering modest doses of IL-2 can enhance the population of Tregs, decrease disease activity, and enhance survival rates [19]. Clinical studies have shown that low-dose IL-2 treatment is linked to an increase in the number and effectiveness of Treg cells, a decrease in disease activity, and a positive safety profile [20]. The results indicate that administering modest doses of IL-2 might be a beneficial addition to the treatment options for SLE. This method specifically targets the immune system and has the potential to provide long-term management of the condition.

## Review

Molecular mechanisms of IL-2 therapy in SLE

Interleukin-2 (IL-2) is an essential cytokine that plays a critical role in regulating the immune system. Originally identified as a T-cell growth factor, it is now acknowledged for its wider involvement in immunological regulation, namely in the upkeep and operation of regulatory T cells (Tregs) [1]. IL-2 is mainly synthesized by activated CD4+ T cells and exerts its effects via binding to its receptor, IL-2R, which consists of three subunits: IL-2Rα (CD25), IL-2Rβ (CD122), and IL-2Rγ (CD132) [2]. When IL-2 binds to its receptor, it initiates signaling pathways that affect the growth, survival, and activity of several types of immune cells, such as Tregs, effector T cells, and natural killer (NK) cells [3].

Tregs are a distinct group of T cells that have a crucial function in preserving immunological tolerance and inhibiting autoimmune reactions. They exhibit elevated levels of CD25, which is the α subunit of the IL-2 receptor, rendering them extremely responsive to IL-2. Tregs depend on IL-2 for their proliferation, viability, and immunosuppressive activity [4]. The binding of IL-2 to IL-2R on Tregs triggers the activation of the Janus kinase-signal transducer and activator of the transcription (JAK-STAT) pathway, namely STAT5. This activation enhances the transcription of genes that are crucial for the formation and functioning of Tregs [5]. In systemic lupus erythematosus (SLE), there is frequently a deficit or malfunction of regulatory T cells (Tregs), which leads to a breakdown in immunological tolerance and the emergence of autoimmunity [6]. The objective of low-dose IL-2 treatment is to specifically increase and stimulate Tregs by taking advantage of their strong attraction to IL-2. This targeted expansion increases the ability of Tregs to inhibit immunological responses, hence restoring immune equilibrium and decreasing autoimmune reactions [7].

IL-2 is essential for the functioning of Treg cells, but it also contributes to the growth and activation of Teff cells. If not appropriately controlled, this might worsen autoimmune reactions. Teffs have reduced levels of CD25 in comparison to Tregs, necessitating larger quantities of IL-2 for their stimulation [8]. This distinct need enables low-dose IL-2 treatment to selectively target Tregs without substantially activating Teffs. By precisely adjusting the dosage of IL-2, it is feasible to increase the number of Tregs while reducing the activation of Teffs, thereby facilitating immunological tolerance without intensifying autoreactivity [9].

IL-2 exerts its effects via binding to the IL-2R, which triggers several signaling pathways such as the JAK-STAT system, the phosphatidylinositol 3-kinase (PI3K)-protein kinase B (Akt) route, and the mitogen-activated protein kinase (MAPK) pathway [10]. When IL-2 binds to IL-2Rα, it causes JAK1 and JAK3 to be brought to the receptor complex. This leads to the phosphorylation and activation of STAT5. Upon activation, STAT5 migrates to the nucleus, where it facilitates the transcription of genes that are crucial for the proliferation, survival, and functioning of Treg cells [11]. The PI3K-Akt pathway, which is likewise stimulated by IL-2 signaling, facilitates cell survival and proliferation by suppressing apoptotic pathways and augmenting cellular metabolism. This mechanism plays a crucial role in preserving Tregs and their ability to resist apoptosis [12]. The MAPK pathway, which plays a role in the process of cell differentiation and proliferation, also plays a part in the impact of IL-2 on distinct immune cells [13].

**Figure 1:**
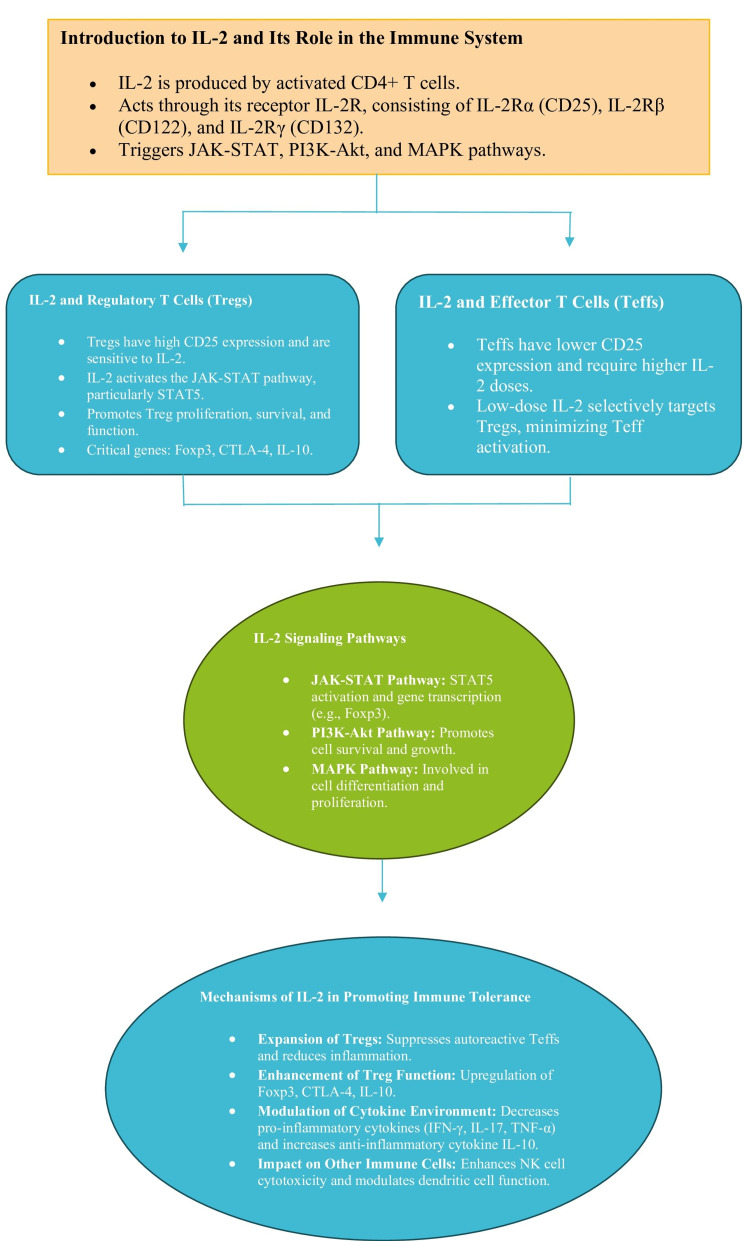
Diagrammatic presentation of the molecular mechanisms of IL-2 therapy in promoting immune tolerance in SLE SLE: Systemic Lupus Erythematosus, IL-2: Interleukin-2, CD4+: Cluster of Differentiation 4 Positive, IL-2R: Interleukin-2 Receptor, IL-2Rα: Interleukin-2 Receptor Alpha, CD25: Cluster of Differentiation 25, IL-2Rβ: Interleukin-2 Receptor Beta, CD122: Cluster of Differentiation 122, IL-2Rγ: Interleukin-2 Receptor Gamma, CD132: Cluster of Differentiation 132, JAK-STAT: Janus Kinase-Signal Transducer and Activator of Transcription, PI3K-Akt: Phosphoinositide 3-Kinase-Akt, MAPK: Mitogen-Activated Protein Kinase, Tregs: Regulatory T Cells, STAT5: Signal Transducer and Activator of Transcription 5, Foxp3: Forkhead Box Protein P3, CTLA-4: Cytotoxic T-Lymphocyte-Associated Protein 4, IL-10: Interleukin-10, Teffs: Effector T Cells, IFN-γ: Interferon Gamma, IL-17: Interleukin-17, TNF-α: Tumor Necrosis Factor Alpha, NK: Natural Killer Image credits: Gurjot Singh

The primary mechanism in which low-dose IL-2 increases immunological tolerance is by specifically increasing the number of Tregs (regulatory T cells). The augmentation of regulatory T cell (Treg) populations leads to a heightened ability to regulate autoreactive effector T cells (Teffs), resulting in a decrease in autoimmune inflammation and tissue damage in SLE [14]. IL-2 not only augments the quantity of Tregs but also amplifies their inhibitory capacity. This is accomplished by increasing the expression of genes that are involved in the suppression of Treg cells, such as forkhead box P3 (Foxp3), cytotoxic T-lymphocyte-associated protein 4 (CTLA-4), and IL-10 [15]. Foxp3 is an essential transcription factor for the formation and functioning of regulatory T cells (Tregs), whereas CTLA-4 and IL-10 play crucial roles in the suppressive activity of Tregs [16]. IL-2 treatment alters the cytokine environment by decreasing the production of pro-inflammatory cytokines such as interferon (IFN)-γ, IL-17, and tumor necrosis factor (TNF)-α, which are increased in SLE [17]. Simultaneously, IL-2 stimulates the synthesis of anti-inflammatory cytokines such as IL-10, therefore enhancing the process of inflammation resolution (Figure 1) [18].

IL-2 not only affects Tregs and Teffs but also has an impact on other immune cells, such as NK cells and dendritic cells. Interleukin-2 (IL-2) has the ability to increase the cytotoxic activity of natural killer (NK) cells and regulate the antigen-presenting function of dendritic cells, so promoting a more controlled immune response [19]. Additionally, Sharabi et al. have explored novel treatment methods for SLE that further highlight the broad potential of IL-2 in modulating immune responses (Table 1) [21].

**Table 1:** Molecular mechanisms of low-dose IL-2 therapy in SLE SLE: Systemic Lupus Erythematosus, Tregs: Regulatory T cells, IL-2: Interleukin-2, IL-2R: Interleukin-2 receptor, JAK1: Janus kinase 1, JAK3: Janus kinase 3, STAT5: Signal transducer and activator of transcription 5, PI3K: Phosphoinositide 3-kinase, Akt: Protein kinase B, MAPK: Mitogen-activated protein kinase, Foxp3: Forkhead box P3, CTLA-4: Cytotoxic T-lymphocyte-associated protein 4, IL-10: Interleukin-10, IFN-γ: Interferon-gamma, IL-17: Interleukin-17, TNF-α: Tumor necrosis factor-alpha, NK cells: Natural killer cells

Molecular Mechanism	Description
Regulation of Tregs	IL-2 selectively expands and activates regulatory T cells (Tregs), which maintain immune tolerance.
JAK-STAT Pathway	IL-2 binds to IL-2R, activating JAK1 and JAK3, leading to STAT5 phosphorylation and promotion of Treg function.
PI3K-Akt Pathway	Enhances Treg survival and resistance to apoptosis, promoting cell survival and metabolic activity.
MAPK Pathway	Contributes to Treg proliferation and differentiation.
Enhancement of Treg Function	IL-2 increases expression of genes like Foxp3, CTLA-4, and IL-10, enhancing Treg suppressive activity.
Modulation of Cytokine Environment	Reduces pro-inflammatory cytokines (IFN-γ, IL-17, TNF-α) and promotes anti-inflammatory cytokines (IL-10).
Impact on Effector T Cells	Preferentially targets Tregs without significantly stimulating effector T cells, minimizing autoreactivity.
Influence on NK Cells and Dendritic Cells	Enhances NK cell cytotoxic activity and modulates dendritic cell antigen-presenting function.

Discussion

Systemic lupus erythematosus (SLE) is an autoimmune illness that is difficult to treat because of its intricate development and diverse clinical characteristics. Present treatments frequently include extensive immunosuppression, which might result in notable adverse effects and may not consistently provide sufficient disease management. Within this particular framework, the utilization of low-dose IL-2 treatment has arisen as a potentially advantageous method because of its capacity to specifically increase the population of regulatory T cells (Tregs) and regulate immune responses. This discussion provides a thorough analysis of current research and clinical studies that have investigated the effectiveness and safety of low-dose IL-2 treatment in SLE. It also addresses the various pathways that contribute to its therapeutic benefits.

Interleukin-2 (IL-2) was identified as a substance that promotes the proliferation of T-cells in the 1970s. Its function in regulating the immune system has been thoroughly investigated since then. Originally employed in high dosages for the treatment of malignancies, specifically metastatic melanoma and renal cell carcinoma, IL-2 therapy had notable immunomodulatory properties [22]. Nevertheless, the significant adverse effects observed at elevated dosages have restricted its wider utilization [23]. Belimumab, Rituximab, and Anifrolumab, which are now employed in the treatment of SLE, selectively act on distinct elements of the immune system. Belimumab hinders the action of the B-cell activating factor (BAFF), hence decreasing the survival of B-cells and the formation of autoantibodies [12]. Rituximab specifically targets CD20 molecules on B cells, resulting in the removal of B cells from the body [13]. Anifrolumab specifically targets the receptor for type I interferon, therefore blocking the interferon pathway that is involved in the development of systemic lupus erythematosus (SLE) [14]. Although these medicines are successful, they can lead to substantial immunosuppression and an elevated risk of infection [15].

IL-2 is crucial in regulating the immune system by increasing the number and activity of Tregs. Tregs have elevated expression of the IL-2 receptor alpha chain (CD25), rendering them very responsive to IL-2 [4]. IL-2 interaction with its receptor triggers many pathways, including the JAK-STAT pathway, which enhances the transcription of genes crucial for Treg formation and function [5]. The PI3K-Akt pathway promotes the survival of Treg cells and makes them more resistant to apoptosis. On the other hand, the MAPK pathway plays a role in the proliferation and differentiation of Treg cells [13]. Low-dose IL-2 treatment has been demonstrated in clinical studies to successfully enhance the numbers of Treg cells and improve clinical outcomes in patients with systemic lupus erythematosus (SLE) [10,11,20]. Nevertheless, the diversity of SLE results in substantial variations in patients' reactions. Baseline disease activity, genetic background, and past therapies can impact the effectiveness of IL-2 therapy.

The safety profile of low-dose IL-2 treatment is excellent, with moderate injection site responses and temporary flu-like symptoms being the most prevalent side effects [10]. When compared to other biologics, which can lead to significant weakening of the immune system and higher chances of being infected, low-dose IL-2 mainly improves the activity of Treg cells without causing a widespread suppression of the immune system [15,20]. Gaining insight into the reasons for the lack of response to low-dose IL-2 treatment in certain individuals is of utmost importance. Possible causes of resistance may involve Treg dysfunction, changes in IL-2 receptor expression, and variations in downstream signaling pathways [16]. Additional investigation is required to ascertain biomarkers that have the ability to forecast the response to IL-2 treatment.

Although short-term studies suggest that low-dose IL-2 is both safe and effective, there is a lack of long-term evidence available. Possible hazards encompass the formation of neutralizing antibodies against IL-2 and disruptions in immunological homeostasis [17]. Conducting long-term follow-up studies is crucial in order to thoroughly evaluate the safety and long-lasting effectiveness of IL-2 treatment. Low-dose IL-2 treatment provides a focused method of adjusting the immune system, which may decrease the requirement for widespread immune suppression and the accompanying expenses. Nevertheless, it is important to take into account the expense of IL-2 therapy and the requirement for frequent administration. Further research is needed to compare the cost-effectiveness of IL-2 therapy with other therapies [18].

It is crucial to identify biomarkers on Treg cells that may be used to anticipate how a patient will respond to IL-2 therapy in order to optimize the effectiveness of the treatment. Possible indicators consist of initial Treg levels, expression of IL-2 receptors, and certain genetic markers [19]. Incorporating biomarker studies into clinical practice might assist in customizing IL-2 treatment for specific patients. Continued research is being conducted to enhance the efficiency of IL-2 dosage schedules. Increasing the dosage and exploring different ways of administering the treatment, such as through the mouth or nose, might potentially improve the effectiveness of the therapy and make it more convenient for patients [24]. The therapeutic benefits of low-dose IL-2 may be enhanced by combining it with other immunomodulatory drugs. For example, the combination of IL-2 with rituximab or belimumab has the potential to produce synergistic advantages by targeting several components of the immune response [13]. It is necessary to conduct clinical studies to investigate these combinations.

The diverse nature of SLE requires an individualized approach to therapy. By incorporating IL-2 therapy within a precision medicine framework, tailoring the treatment based on a patient’s unique genetic and immune characteristics. This approach aims to enhance the effectiveness of IL-2 therapy by selecting the right patients, optimizing dosing, combining with other therapies, and continuously monitoring response, leading to better outcomes and fewer side effects [24]. Obtaining regulatory approval and addressing ethical issues are essential aspects of the development process for novel IL-2 formulations. To guarantee the safety and effectiveness of these novel formulations, it is imperative to conduct extensive studies involving several centers. Additionally, ethical concerns about patient selection and treatment methods must be carefully addressed [25].

Utilizing low-dose IL-2 treatment has great potential as a novel method to regulate the immune system in patients with systemic lupus erythematosus (SLE). The capacity to specifically increase and activate Tregs addresses a crucial deficiency in the development of SLE, providing a focused and perhaps safer alternative to traditional immunosuppressive treatments. The treatment has been extensively studied through clinical trials and mechanistic investigations, which have provided strong evidence of its effectiveness and safety. Ongoing research is focused on improving its usage and gaining a deeper knowledge of its long-term consequences. Incorporating low-dose IL-2 therapy into the treatment strategy for SLE has the capacity to greatly enhance patient outcomes by offering a more accurate and individualized method of immune regulation. Future research should prioritize the improvement of dosage regimens, the identification of biomarkers for patient selection, and the investigation of combination medicines to augment therapeutic success. By tackling these obstacles and leveraging the existing data, the use of low-dose IL-2 therapy has the potential to become a fundamental component in the treatment of SLE and other autoimmune disorders [26].

The safety profile of low-dose IL-2 therapy is an important factor to consider when evaluating its potential as a treatment for SLE. Low-dose IL-2 has been regularly found to be well-tolerated in clinical studies, with little occurrence of adverse effects. Typical negative effects include moderate responses at the injection site and temporary flu-like symptoms, which are usually controllable and do not result in stopping the treatment [10]. The low-dose IL-2 has a good safety profile due to its specific method of action. Low-dose IL-2 selectively targets Tregs, which play a vital role in immunological control, therefore avoiding the general suppression of the immune system that is commonly linked with traditional treatments. This selectivity decreases the likelihood of infections and other problems that are frequently observed with high-dose immunosuppressive therapies [11].

Low-dose IL-2 has distinct advantages over other biologic therapies for SLE, such as belimumab and rituximab. Belimumab, an antibody that inhibits BAFF, and rituximab, an antibody that targets CD20, both specifically work on B cells to decrease the formation of autoantibodies and mitigate disease activity. Although these medications can be beneficial for certain individuals, they can also cause substantial immunosuppression and a heightened susceptibility to infections [4,5]. Low-dose IL-2 treatment specifically boosts Treg function without causing widespread suppression of the immune system. This mechanism of action offers a more focused method of immune regulation, which might potentially decrease the likelihood of negative consequences linked to widespread immunosuppression. In addition, IL-2 treatment may be delivered subcutaneously, which is less intrusive compared to the intravenous administration necessary for several biologics [13].

The unique mechanism of low-dose IL-2 therapy also offers possibilities for combination therapies. The combination of IL-2 with other immunomodulatory drugs might potentially improve the effectiveness of therapy by addressing many components of the immune response simultaneously. For instance, the combination of low-dose IL-2 with rituximab may result in synergistic benefits by concurrently decreasing B cell activity and augmenting Treg-mediated suppression [13]. In addition, IL-2 therapy has the potential to be used in conjunction with current standard-of-care medications to enhance illness management. Further research is needed to investigate the effectiveness of combining low-dose IL-2 with standard immunosuppressive medications, such as mycophenolate mofetil or hydroxychloroquine, in order to identify the most effective treatment methods for patients with systemic lupus erythematosus (SLE) [15]. Previous research has demonstrated the efficacy of IL-2 in restoring regulatory T-cell homeostasis in other conditions, such as graft-versus-host disease, providing further evidence of its potential in combination therapies [22]. Current research is actively investigating the possibilities of low-dose IL-2 treatment in SLE and other autoimmune illnesses. Multiple ongoing clinical trials are presently being conducted to better examine the effectiveness, safety, and most suitable dosage schedules of the treatment. The objective of this research is to enhance the therapeutic procedures for IL-2 therapy and determine the specific patient populations who are most likely to see positive outcomes from this treatment. A promising field of study is the advancement of IL-2 variants or fusion proteins that have been modified to improve their pharmacokinetics and increase their effectiveness as treatments. The purpose of these altered versions of IL-2 is to enhance the amount of the drug that can be utilized by the body, decrease the number of times it needs to be administered, and lessen the likelihood of an immune response [15]. These developments have the potential to enhance the practicality of IL-2 treatment in treating SLE. Furthermore, the crucial objective is to identify biomarkers that may accurately predict the response to IL-2 treatment. Baseline Treg levels, IL-2 receptor expression, and particular genetic markers are biomarkers that may be used to customize IL-2 therapy for each patient, leading to better results and minimizing needless medication [24].

Two extensive studies have greatly contributed to our understanding of the effectiveness and safety of low-dose IL-2 treatment for SLE, leading to recent developments in this field. The first study conducted by Su et al. (2024) provides a comprehensive evaluation and statistical analysis of the effects of low-dose IL-2 treatment on several autoimmune rheumatic illnesses, such as SLE. This meta-analysis provides evidence that the administration of low-dose IL-2 effectively enhances the number of Treg cells and promotes a favorable change in the Th17/Tregs ratio. This shift is essential for the restoration of immunological tolerance in autoimmune diseases [25]. Although the study's wide range of subjects increases the applicability of its conclusions, it also raises questions regarding the accuracy of these results for SLE. This is because the inclusion of data from different autoimmune disorders may hide the special details related to SLE therapy. However, the results of the study provide evidence for the effectiveness of low-dose IL-2 as a flexible treatment for regulating the immune system, further emphasizing its importance in the overall management of autoimmune diseases [25].

On the other hand, the second research conducted by Su et al. (2024) specifically examines the impact of low-dose IL-2 on SLE, offering a more focused analysis that directly addresses the treatment requirements of SLE patients. This study highlights the effectiveness of the medication in increasing the status of Treg cells, lowering disease activity, and boosting clinical outcomes specifically for systemic lupus erythematosus (SLE) [26]. The results are consistent with prior studies but also highlight the diversity in how patients respond, highlighting the need to tailor SLE treatment to each individual. Significantly, this study emphasizes the advantageous safety characteristics of low-dose IL-2, exhibiting little occurrence of negative consequences. This positions it as a possible substitute for conventional immunosuppressive treatments. The study's emphasis on outcomes unique to systemic lupus erythematosus (SLE) offers valuable insights that might inform more accurate and personalized treatment approaches, thereby enhancing patient care and long-term results [26].

Notwithstanding the encouraging outcomes, there are other constraints and difficulties that must be tackled. An important obstacle lies in the inconsistency of patient reactions to low-dose IL-2 treatment. While a considerable number of individuals derive substantial therapeutic advantages, there are some who exhibit limited or no enhancement. Comprehending the mechanisms that contribute to this variability is essential for maximizing therapy effectiveness [27]. Furthermore, a comprehensive understanding of the long-term consequences of IL-2 treatment is still lacking. Although short-term trials suggest a positive safety profile, it is necessary to get long-term data in order to evaluate the possible dangers and advantages of chronic IL-2 treatment. It is crucial to monitor for any negative effects, such as the formation of neutralizing antibodies against IL-2 or changes in immunological balance, in order to ensure the safety of this treatment [28]. Another factor to take into account is the possibility of IL-2 treatment unintentionally stimulating harmful effector T cells in certain individuals, which might worsen the condition. While low-dose IL-2 primarily increases the number of Tregs, it is crucial to carefully manage the equilibrium between Tregs and effector T cells to prevent any unwanted outcomes [22].

## Conclusions

Utilizing low-dose IL-2 treatment has great potential as a novel method to regulate the immune system in individuals with SLE. The capacity to specifically increase and activate Tregs addresses a crucial deficiency in the development of SLE, providing a focused and perhaps less risky alternative to traditional immunosuppressive treatments. The effectiveness and safety of this medication are well-supported by clinical trials and mechanistic investigations, with current research focused on optimizing its usage and comprehending its long-term implications. Incorporating low-dose IL-2 therapy into the treatment strategy for SLE has the capacity to greatly enhance patient outcomes by offering a more accurate and individualized method for immune regulation. Future research should prioritize the improvement of dosage regimens, the identification of biomarkers for patient selection, and the investigation of combination medicines to augment therapeutic success. By tackling these difficulties and leveraging the existing data, the use of low-dose IL-2 therapy has the potential to become a fundamental component in the treatment of SLE and other autoimmune disorders.
